# Age cutoff for Epstein-Barr virus-positive diffuse large B-cell lymphoma–is it necessary?

**DOI:** 10.18632/oncotarget.4324

**Published:** 2015-05-29

**Authors:** Chi Young Ok, Qing Ye, Ling Li, Ganiraju C. Manyam, Lijuan Deng, Rashmi R. Goswami, Xiaoxiao Wang, Santiago Montes-Moreno, Carlo Visco, Alexandar Tzankov, Karen Dybkaer, Li Zhang, Jeremy Abramson, Aliyah R. Sohani, April Chiu, Attilio Orazi, Youli Zu, Govind Bhagat, Kristy L. Richards, Eric D. Hsi, William W.L. Choi, J. Han van Krieken, Jooryung Huh, Maurilio Ponzoni, Andrés J.M. Ferreri, Shanxiang Zhang, Ben M. Parsons, Mina Xu, Michael B. Møller, Jane N. Winter, Miguel A. Piris, Zijun Y. Xu-Monette, L. Jeffrey Medeiros, Ken H. Young

**Affiliations:** ^1^ Department of Hematopathology, The University of Texas MD Anderson Cancer Center, Houston, Texas, USA; ^2^ Department of Bioinformatics and Computational Biology, The University of Texas MD Anderson Cancer Center, Houston, Texas, USA; ^3^ Hospital Universitario Marques de Valdecilla, Santander, Spain; ^4^ San Bortolo Hospital, Vicenza, Italy; ^5^ University Hospital, Basel, Switzerland; ^6^ Aalborg University Hospital, Aalborg, Denmark; ^7^ Massachusetts General Hospital and Harvard Medical School, Boston, MA, USA; ^8^ Memorial Sloan-Kettering Cancer Center, New York, New York, USA; ^9^ Weill Medical College of Cornell University, New York, New York, USA; ^10^ Houston Methodist Hospital, Houston, Texas, USA; ^11^ Columbia University Medical Center and New York Presbyterian Hospital, New York, New York, USA; ^12^ University of North Carolina School of Medicine, Chapel Hill, North Carolina, USA; ^13^ Cleveland Clinic, Cleveland, Ohio, USA; ^14^ University of Hong Kong Li Ka Shing Faculty of Medicine, Hong Kong, China; ^15^ Radboud University Nijmegen Medical Centre, Nijmegen, the Netherlands; ^16^ Asan Medical Center, Ulsan University College of Medicine, Seoul, Korea; ^17^ San Raffaele H. Scientific Institute, Milan, Italy; ^18^ Indiana University School of Medicine, Indianapolis, Indiana, USA; ^19^ Gundersen Lutheran Health System, La Crosse, Wisconsin, USA; ^20^ Yale University, School of Medicine, New Haven, CT, USA; ^21^ Odense University Hospital, Odense, Denmark; ^22^ Feinberg School of Medicine, Northwestern University, Chicago, Illinois, USA; ^23^ The University of Texas School of Medicine, Graduate School of Biomedical Sciences, Houston, Texas, USA

**Keywords:** EBV, DLBCL, elderly, gene expression profiling

## Abstract

Epstein-Barr virus-positive diffuse large B-cell lymphoma of the elderly (EBV+ DLBCL-e) is a molecularly distinct variant of DLBCL, characterized by a monoclonal B-cell proliferation that occurs in patients >50 years of age without a history or clinicopathologic evidence of immunodeficiency. However, patients with EBV+ DLBCL younger than 50-years-old also exist in Western countries. We evaluated the clinicopathologic, immunophenotypic and genetic features in Cacausian patients with EBV+ DLBCL who are ≤50 years of age and compared this patient group to patients who are >50 years. In patients who are ≤50 years, less frequent expression of BCL6 and a trend of more frequent expression of CD30 and pSTAT3 were found in patients with EBV+ DLBCL. In patients who are >50 years, common expression of CD30, p50, pSTAT3 and less frequent expression of BCL6 were observed. Older patients also more commonly had a poor performance status (ECOG≥2). Comparing EBV+ DLBCL patients in ≤50 years versus >50 years, both groups had similar clinicopathologic, immunophenotypic and genetic features. Gene expression profiling, microRNA profiling and treatment outcome of the younger patients with EBV+ DLBCL was not distinctive from tumors in older patients. Based on our data, we suggest that the arbitrary age cutoff for EBV+ DLBCL is unnecessary and should be eliminated in the WHO lymphoma classification scheme.

## INTRODUCTION

Epstein-Barr virus (EBV) positive diffuse large B-cell lymphoma of the elderly (EBV+ DLBCL-e) is a monoclonal B-cell lymphoid proliferation that occurs in patients > 50 years without evidence of immunodeficiency or a history of lymphoma [1]. EBV+ DLBCL-e constitutes 8-10% and 2-5% of DLBCL in Asian countries and Western countries, respectively [2-5]. EBV+ DLBCL-e is a molecularly distinct entity characterized by enhanced activity of the NF-κB, signal transducer and activator of transcription 3 (STAT3), MEK/ERK and phosphoinositide 3-kinase (PI3K)/Akt pathways, mostly induced by EBV products [6]. In the era of treatment with cyclophosphamide, doxorubicin, vincristine and prednisone (CHOP), patients with EBV+ DLBCL-e were thought to be an aggressive variant of DLBCL. With the current therapeutic regimen, CHOP plus rituximab (R-CHOP), EBV+DLBCL does not confer a worse prognosis in Western patients [5, 7]. However, the data is controversial in Asian patients [8, 9].

The median age of patients with EBV+ DLBCL-e is 71 years and the prevalence of EBV positivity in DLBCL increases with age, as high as 30% in patients > 90 years [10]. The current World Health Organization (WHO) classification sets an arbitrary age cutoff of 50 years as a defining feature of EBV+ DLBCL-e. However, well-documented cases of DLBCL with EBV infection in apparently immunocompetent young adults or even in children have been reported, questioning the rationale of the current age cutoff [11-13]. To the best of our knowledge, Hong et al has been the only group of investigators who systematically compared EBV positivity in young (≤50 years) versus old (> 50 years) patients with DLBCL [12]. EBV infection was less common in younger compared with older patients (6.7% *vs*. 9.3%). In younger, patients with EBV+ DLBCL did not have distinct clinical features or worse outcome compared with patients with EBV-negative DLBCL. In the elderly group, however, EBV positivity was correlated with advanced stage, high IPI risk group (age-adjusted), and involvement of ≥2 extranodal sites. The older patients with EBV+ DLBCL also showed shorter overall survival and progression-free survival compared with young patients. These authors also showed that EBV positivity was an independent risk factor for overall survival in R-CHOP treated elderly patients. In aggregate, the data presented by Hong et al appears to support the age cutoff in the WHO classification.

In this study, our aim was to further compare clinicopathologic, immunophenotypic, and molecular findings of young (≤50 years) versus older (> 50 years) patients with EBV+ DLBCL.

## RESULTS

### EBV infection occurs in all age groups and similar morphologic variants observed between both younger and elderly groups

**Figure 1:**
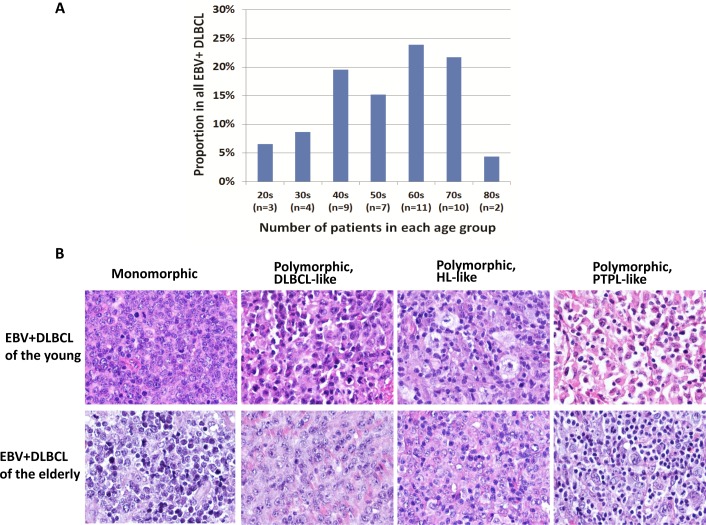
Distribution and morphologic variants of EBV+ DLBCL patients in each age group **A.** Distribution of EBV+ DLBCL patients in each age group. **B.** Morphologic variants in EBV-positive diffuse large B-cell lymphoma of the young (upper panel) and elderly (lower panel). Similar variants are seen in both groups of patients. The monomorphic subtype is featured by monotonous sheets of large transformed B cells. The polymorphic DLBCL-like subtype shows canonical large B-cell neoplasm morphology, with a high density of large neoplastic cells and scattered cells with (Reed-Sternberg) RS-like and Hodgkin-like features. The polymorphic HL-like subtype displays a lower density of neoplastic cells with RS-like and Hodgkin-like features. The polymorphic LPD-like subtype is a DLBCL with polymorphic lymphoproliferative disorder (LPD)-like features. It is characterized by a low density of neoplastic cells without HL-like features.

A total of 46 cases of EBV+ DLBCL were identified. The number of EBV+ DLBCL patients in different age group is shown in Figure 1A. There were 16 (35%) and 30 (65%) patients in the younger (≤50 years) and older (> 50 years) group, respectively. Similar morphologic variants were seen in the younger (≤50 years) and older (> 50 years) group (Figure 1B). The monomorphic subtype is featured by monotonous sheets of large transformed B cells. The polymorphic DLBCL-like subtype shows canonical large B-cell neoplasm morphology, with a high density of large neoplastic cells and scattered cells with (Reed-Sternberg) RS-like and Hodgkin-like features. The polymorphic HL-like subtype displays a lower density of neoplastic cells with RS-like and Hodgkin-like features. The polymorphic LPD-like subtype is a DLBCL with polymorphic lymphoproliferative disorder (LPD)-like features. It is characterized by a low density of neoplastic cells without HL-like features. In both age groups, EBV+ DLBCL patients did not show differences in their clinical parameters. Nineteen (58%) cases had an ABC phenotype and 14 (42%) cases had a GCB phenotype. Expression of LMP1 and EBNA2 was found 17 (68%) and 5 (22%) patients, respectively. Expression of CD30 (44% *vs*. 14%, *p* < 0.0001), p50 (61% *vs*. 34%, *p* = 0.0124) and phosphorylated signal transducer and activator of transcription 3 (pSTAT3) (39% *vs*. 16%, *p* = 0.0079) were more commonly observed than the EBV negative de novo DLBCL patients. BCL6 expression (50% *vs*. 82%, *p* = 0.002) was less commonly observed in EBV+ DLBCL. Rearrangements of *BCL2, BCL6* or *MYC* and *TP53* mutation were infrequent in this study group.

### Effect of EBV infection in patients ≤50 years of age

**Figure 2:**
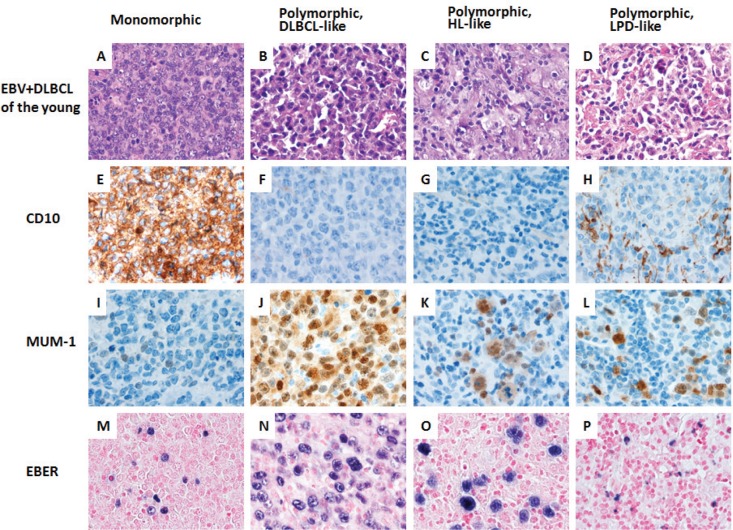
A spectrum of morphologic variants and immunophenotypic profiling in EBV-positive diffuse large B-cell lymphoma of the younger patients **A. E. I. M.**, The monomorphic case presented in this figure shows the GCB subtype. All polymorphic subtypes show the ABC-DLBCL molecular phenotype. **B. F. J. N.**, Polymorphic DLBCL-like variant shows canonical large B-cell neoplasm morphology. **C. G. K. O.**, The polymorphic HL-like variant shows Hodgkin lymphoma-like features. **D. H. L. P.**, The polymorphic LPD-like variant shows polymorphic lymphoproliferative disorder (LPD)-like features with a low density of neoplastic cells without HL-like features. All images are shown at a magnification of x 600.

Morphologic analysis showed the monomorphic variant in 3 (18%) and the polymorphic variants in 13 (82%) tumors (Figure 2). In younger patients, EBV+ DLBCL did not show distinct clinicopathologic features compared with DLBCL without EBV infection in younger patients (Table 1). In younger patients with EBV+ DLBCL, patients with an ABC (55%) phenotype was slightly more common than a GCB phenotype (45%) (*p* = 0.1793). Expression of LMP1 and EBNA2 was found 4 (67%) and 2 (40%) patients, respectively. BCL6 expression (38%) was significantly lower in EBV+ DLBCL (*p* = 0.016). Expression of CD30 (43%) and pSTAT3 (43%) were more commonly observed, but did not achieve statistical significance (*p* = 0.0727 and *p* = 0.0818, respectively). There was no significant difference in expression of other markers. Rearrangements in *BCL2, BCL6* and *MYC* and *TP53* mutation were infrequently detected in patients < 50 years with EBV+ DLBCL.

**Table 1:** Clinical, immunophenotypic and genetic features of EBV+ DLBCL and EBV− DLBCL

	All patients			≤50 years			>50 years		
	n=46	n=533	P value	n=16	n=104	P value	n=30	n=429	P value
	EBV+ DLBCL	EBV− DLBCL		EBV+ DLBCL	EBV− DLBCL		EBV+ DLBCL	EBV− DLBCL	
**Median age, years (range)**	61 (21 - 86)	63 (16 - 92)	0.1481	43 (21-50)	42 (16-50)	0.8161	66 (53-86)	51 (67 - 92)	0.6916
**Men:women**	25:21	306:227	0.7567	7:9	69:35	0.1581	18:12	237:192	0.7054
**B symptoms**	16 (38%)	154 (33%)	0.4981	7 (47%)	29 (31%)	0.2248	9 (33%)	125 (33%)	1
**LDH elevation**	31 (70%)	304 (63%)	0.4133	12 (75%)	65 (69%)	0.5458	19 (68%)	239 (62%)	0.5525
**Stage III/IV**	28 (62%)	266 (52%)	0.2133	9 (56%)	50 (51%)	0.5869	19 (66%)	216 (52%)	0.1807
**ECOG ≥2**	11 (27%)	72 (16%)	0.0783	2 (13%)	11 (13%)	1	9 (36%)	61 (16%)	**0.0251**
**IPI >2**	16 (38%)	221 (42%)	0.6295	2 (13%)	33 (33%)	0.2204	14 (52%)	188 (45%)	0.5506
**≥2 EN involvement**	9 (20%)	114 (22%)	0.8522	3 (19%)	30 (30%)	0.5491	6 (21%)	84 (20%)	0.8139
**Size ≥ 6cm**	12 (36%)	130 (33%)	0.7006	5 (38%)	25 (33%)	0.7447	7 (35%)	105 (32%)	0.8086
**CR/PR**	39 (89%)	471 (88%)	1	12 (86%)	93 (89%)	0.637	27 (90%)	378 (88%)	1
**GCB phenotype**	14 (42%)	280 (54%)	0.2806	5 (45%)	69 (68%)	0.1793	9 (41%)	211 (50%)	0.5131
**ABC phenotype**	19 (58%)	243 (46%)		6 (55%)	32 (32%)		13 (59%)	211 (50%)	
**CD30 expression**	16 (44%)	66 (14%)	**<0.001**	6 (43%)	16 (18%)	0.0727	10 (45%)	50 (14%)	**0.0005**
**p50 expression**	14 (61%)	150 (34%)	**0.0124**	3 (43%)	32 (37%)	1	11 (69%)	118 (33%)	**0.0056**
**p65 expression**	3 (11%)	92 (20%)	0.3252	0 (0%)	24 (28%)	0.1849	3 (15%)	68 (18%)	1
**c-Rel expression**	5 (23%)	103 (23%)	1	1 (14%)	16 (19%)	1	4 (27%)	87 (24%)	0.7673
**pSTAT3 expression**	9 (39%)	65 (16%)	**0.0079**	3 (43%)	11 (14%)	0.0818	6 (38%)	54 (16%)	**0.0389**
**BCL2 expression**	12 (41%)	225 (49%)	0.4485	3 (38%)	33 (37%)	1	9 (43%)	192 (52%)	0.5024
**BCL6 expression**	14 (50%)	417 (82%)	**0.0002**	3 (38%)	76 (80%)	**0.016**	11 (52%)	241 (82%)	**0.0024**
**p53 expression**	10 (43%)	163 (36%)	0.5112	2 (29%)	31 (36%)	1	8 (39%)	132 (36%)	0.8072
**MYC expression**	17 (61%)	293 (64%)	0.6913	3 (43%)	47 (54%)	0.702	14 (67%)	246 (66%)	1
**MYC/BCL2 expression**	8 (29%)	152 (34%)	0.6823	1 (14%)	21 (24%)	1	7 (33%)	131 (36%)	1
***BCL2* rearrangement**	1 (5%)	77 (19%)	0.1456	0 (0%)	11 (14%)	1	1 (6%)	66 (20%)	0.3257
***BCL6* rearrangement**	1 (6%)	119 (34%)	**0.026**	0 (0%)	18 (25%)	0.5676	1 (8%)	101 (36%)	0.0628
***MYC* rearrangement**	2 (11%)	38 (9%)	0.6727	0 (0%)	8 (10%)	1	2 (14%)	30 (8%)	0.3571
*Double hit	0 (0%)	16 (4%)	1	0 (0%)	3 (4%)	1	0 (0%)	13 (4%)	1
***TP53* mutation**	2 (10%)	107 (23%)	0.2715	0 (0%)	18 (20%)	1	2 (17%)	89 (23%)	0.3805

LDH; lactate dehydrogenase, ECOG; Eastern Cooperative Oncology Group, IPI; International Prognostic Index, EN; Extranodal, CR; complete remission, PR; partial remission, GCB; germinal center B-cell-like phenotype, ABC; activated B-cell-like phenotype, pSTAT3; phosphorylated signal transducer and activator of transcription 3.

* Double hit; *MYC* rearrangement with either *BCL2* or *BCL6* rearrangement

### Effect of EBV infection in patients > 50 years of age

**Figure 3:**
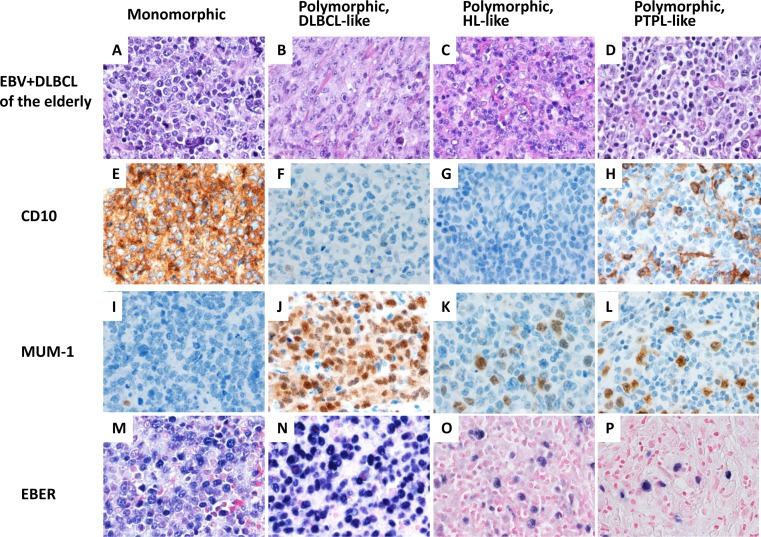
A spectrum of morphologic variants and immunophenotypic profiling in EBV-positive diffuse large B-cell lymphoma of the elderly patients **A. E. I. M.**, The monomorphic case presented in this figure shows the GCB subtype. All polymorphic subtypes show the ABC-DLBCL molecular phenotype. **B. F. J. N.**, Polymorphic DLBCL-like variant shows canonical large B-cell neoplasm morphology. **C. G. K. O.**, The polymorphic HL-like variant shows Hodgkin lymphoma-like features. **D. H. L. P.**, The polymorphic PLPD-like variant shows polymorphic lymphoproliferative disorder (LPD)-like features with a low density of neoplastic cells without HL-like features. All images are shown at a magnification of x 600.

In older patients, monomorphic and polymorphic variants represented 5 (17%) and 25 (83%) of cases, respectively (Figure 3). Performance status (ECOG ≥2) was worse in the older age group (Table 1). There were no significant differences in other clinical features between EBV+ and EBV− DLBCL. The ABC phenotype occurred in 18 (59%) and the GCB phenotype in 12 (41%) tumors. Expression of LMP1 and EBNA2 was found 13 (68%) and 3 (17%) patients, respectively. Expression of CD30 (48%), NF-κB p50 (69%) and pSTAT3 (38%) were more commonly observed in EBV+ DLBCL (*p* = 0.0003, *p* = 0.0056 and *p* = 0.0389, respectively). Expression of BCL6 (52%) was less frequent in EBV+ DLBCL in older patients (*p* = 0.0024). There was no significant difference in expression of other markers. Rearrangements in *BCL2, BCL6* and *MYC* and *TP53* mutation were uncommon in EBV+ DLBCL.

### EBV+ DLBCL in patients ≤50 years *vs*. > 50 years old

We also compared the clinicopathologic, immunophenotypic, and genetic features of EBV+ DLBCL in younger (≤50 years) versus older (> 50 years) patients (Table 2). Significant differences were not observed in other clinical features, protein expression profile or genetic features between younger and older patients with EBV+ DLBCL. We performed gene expression profiling in 5 younger and 20 older patients with EBV+ DLBCL and there was no significant differences in gene expression between the two groups. We also compared the expression of hsa-miR-126, hsa-miR-146a, hsa-miR-146b, hsa-miR-150 and hsa-miR-222 in 2 younger and 7 older patients with EBV+ DLBCL and there were no significant differences in expression of these microRNAs between the two groups (Table 3).

**Table 2:** Comparison between Epstein-Barr virus-positive diffuse large B-cell lymphoma patients age ≤50 years and those age >50 years

	Age ≤50 years (n=16)	Age >50 years (n=30)	P value
**Median age, years (range)**	43 (21-50)	66 (53-86)	**<0.0001**
**Men:women**	7:9	18:12	0.3595
**B symptoms**	7 (47%)	9 (33%)	0.5113
**LDH elevation**	12 (75%)	19 (68%)	0.7385
**Stage III/IV**	9 (56%)	19 (66%)	0.7488
**ECOG ≥2**	2 (13%)	9 (36%)	0.1519
**IPI >2**	2 (13%)	14 (52%)	**0.0203**
**≥2 EN involvement**	3 (19%)	6 (21%)	1
**Size ≥ 6cm**	5 (38%)	7 (35%)	1
**CR/PR**	12 (86%)	27 (90%)	0.6467
**GCB phenotype**	5 (50%)	9 (41%)	0.712
**ABC phenotype**	5 (50%)	13 (59%)	
**CD30 expression**	6 (43%)	10 (45%)	1
**LMP1 expression**	4 (66%)	13 (68%)	1
**EBNA2 expression**	2 (40%)	3 (17%)	0.2907
**p50 expression**	3 (43%)	11 (69%)	0.363
**p65 expression**	0 (0%)	3 (15%)	0.5453
**cREL expression**	1 (14%)	4 (27%)	1
**pSTAT3 expression**	3 (43%)	6 (38%)	1
**BCL2 expression**	3 (38%)	9 (43%)	1
**BCL6 expression**	3 (38%)	11 (52%)	1
**p53 expression**	2 (29%)	8 (39%)	0.6214
**MYC expression**	3 (43%)	14 (67%)	0.3809
**MYC/BCL2 expression**	1 (14%)	7 (33%)	0.6334
***BCL2* rearrangement**	0 (0%)	1 (6%)	1
***BCL6* rearrangement**	0 (0%)	1 (8%)	1
***MYC* rearrangement**	0 (0%)	2 (14%)	1
*Double hit	0 (0%)	0 (0%)	N/A
***TP53* mutation**	0 (0%)	2 (17%)	1

LDH; lactate dehydrogenase, ECOG; Eastern Cooperative Oncology Group, IPI; International Prognostic Index, EN; Extranodal, CR; complete remission, PR; partial remission, GCB; germinal center B-cell-like phenotype, ABC; activated B-cell-like phenotype, pSTAT3; phosphorylated signal transducer and activator of transcription 3, N/A; not available

* Double hit; *MYC* rearrangement with either *BCL2* or *BCL6* rearrangement

**Table 3:** Expression of microRNAs in patients with Epstein-Barr virus-positive diffuse large B-cell lymphoma who are ≤50 years and >50 years old

	≤50 years Median* (range)	>50 years Median* (range)	P value**
**miR-126-3p**	1193 (1157 to 1228)	631 (311 to 2102)	0.5252
**miR-146a-5p**	1109 (554 to 1664)	617 (297 to 2709)	0.9625
**miR-146b-5p**	280 (170 to 390)	247 (118 to 1221)	0.7113
**miR-150-5p**	2223 (2138 to 2308)	877 (352 to 3576)	0.2648
**miR-222-3p**	448 (371 to 525)	458 (151 to 1782)	0.7831

* Number indicates the number of detected probes

** Unpaired t-test, two-sided

### Impact on survival

**Figure 4:**
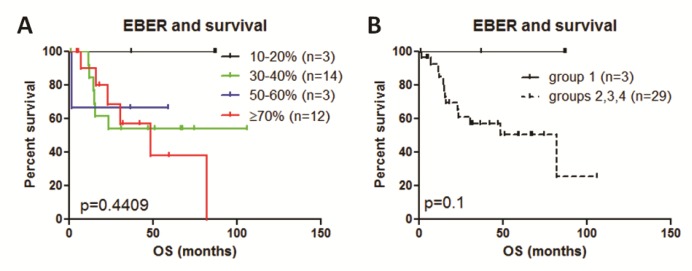
Survival analysis of variable EBER expression impact on survival **A.** Survival analysis of four groups did not show significant difference in survival (*p* = 0.4409) if EBER expression is calculated for group 1 (10-20%), group 2 (30-40%), group 3 (50-60%) and group 4 (≥70%). **B.**, We merged groups 2, 3 and 4 and compared with group 1, but did not show significant difference in overall survival (*p* = 0.1) (**B**).

We first examined if variation in EBER expression dictates significant impact on survival. We created four groups based on EBER expression; group 1 (10-20%), group 2 (30-40%), group 3 (50-60%) and group 4 (≥70%). Survival analysis of these groups did not show significant difference in survival (*p* = 0.4409). (Figure 4A). Although not statistically powered, group 1 appeared to stand out from the remaining groups. We merged groups 2, 3 and 4 and compared with group 1, but did not show significant difference in overall survival (*p* = 0.1) (Figure 4B).

**Figure 5:**
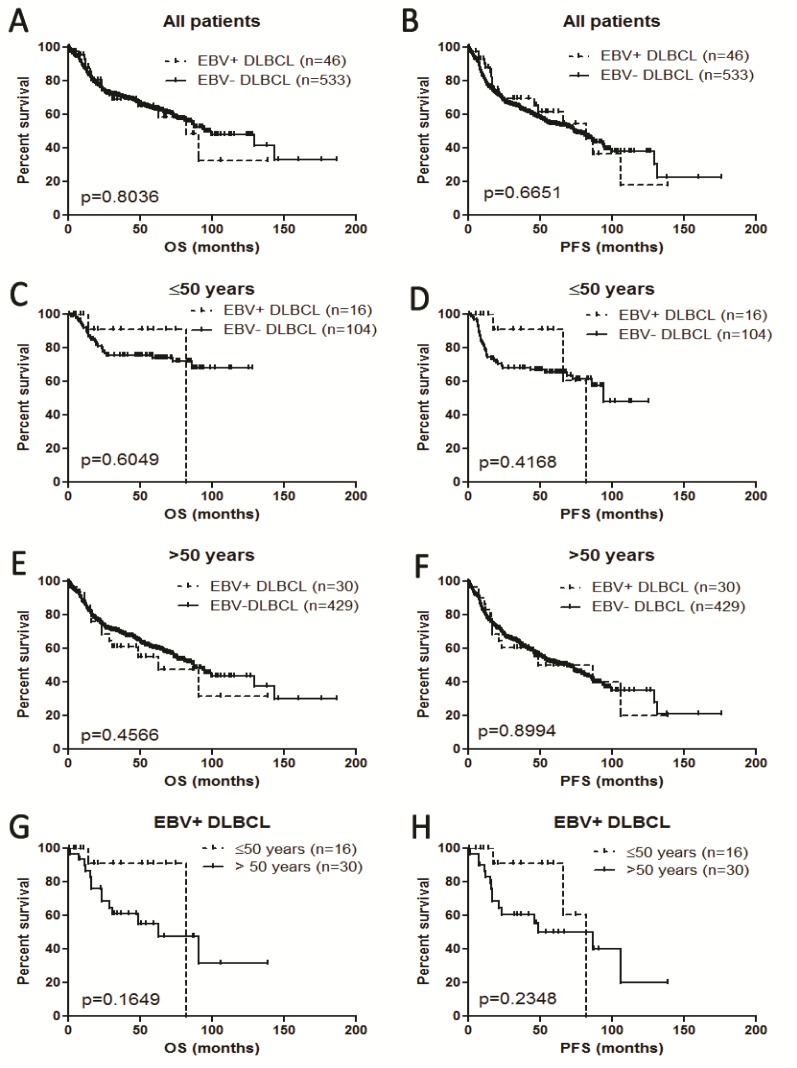
Survival analysis of EBV+ diffuse large B-cell lymphoma in all the patients and different age groups **A.** and **B**. Overall survival (OS) and progression-free survival (PFS) in all patients. **C.** and **D**. OS and PFS in patients ≤50 years. **E**. and **F**. OS and PFS in patients > 50 years. **G.** and **H**. OS and PFS in patients with EBV+ DLBCL.

In all age groups, EBV+ DLBCL versus EBV− DLBCL showed no difference in overall survival (OS) nor progression-free survival (PFS) (Figure 5A and 5B). Separating younger and older patients, EBV+ DLBCL did not show poorer outcome compared with younger and older patients with EBV− DLBCL (Figures 5C, 5D, 5E and 5F). In the group of patients with EBV+ DLBCL, younger (≤50 years) and older (> 50 years) did not show significant differences in OS and PFS (Figure 5G and 5H).

Our group previously identified that EBV+ DLBCL with co-expression of CD30 harbor extremely poor survival [5]. In all age groups, EBV+ DLBCL with co-expression of CD30 had significantly poor overall survival compared with EBV+ DLBCL without CD30 expression (*p* = 0.0382). Separating younger and older patients, however, CD30 co-expression was not statistically powered in overall survival in both groups (*p* = 0.1573 and *p* = 0.1911, respectively).

In all age groups, univariate analysis showed that an increased hazard was observed for the presence of B symptoms, elevated serum LDH, advanced (III/IV) stage, involvement of ≥2 extranodal sites, ECOG ≥2, IPI ≥3, size ≥6 cm and ABC subtype. However, EBV was not associated with increased hazard. Separating younger and older age groups, EBV did not portend increased risk in both groups by univariate analysis. Multivariate analysis was not performed for EBV because univariate analysis was insignificant.

## DISCUSSION

EBV+ DLBCL-e is a provisional entity in the current WHO classification. The notion that DLBCL associated with EBV could harbor a worse outcome has been generally accepted among pathologists and oncologists, but for the age cutoff of 50 years there has been some degree of resistance in the community. The resistance is partly due to the fact the age of 50 years seems arbitrary. EBV+ DLBCL has been documented in patients younger than 50 years of age and the 50 year cutoff seems too young to be designated as “elderly”. Although the age cutoff appears somewhat arbitrary, the data of Oyama and colleagues might provide a possible rationale. In their study, there was a stark difference with respect to the incidence of EBV+ lymphoproliferative disorder in patients without predisposing immunodeficiency in < 50 years (6%) and ≥50 years (94%) [10]. However, our cohort does not reproduce the difference (35% *vs*. 65%, respectively). Instead, there were more patients in the fifth as compared with the sixth decade. The discrepancy between the data of Oyama and colleagues versus the patients group currently presented could be attributable to different ethnic group.

Regardless of age, we did not observe distinct clinical manifestations between patients with EBV+ DLBCL compared with DLBCL without EBV infection. Our data is in contrast with a report from Korea, in which advanced (III/IV) stage, ≥2 extranodal sites of involvement, and high intermediate/high IPI were more common in patients who were > 50 years [12]. The difference also could be attributable to different ethnicities (Korean *vs*. Caucasian). In an earlier study, we showed that EBV+ DLBCL in Caucasian patients is distinct from Asian patients [5]. By immunohistochemistry, expression of CD30, NF-κB p50, pSTAT3 were more frequent and BCL6 expression was less commonly seen in EBV+ DLBCL. Rearrangements of *BCL2, BCL6* or *MYC* and *TP53* mutation were uncommon. These results are consistent with previously published data [5]. In the younger (≤50 years) group, EBV+ DLBCL did not show distinct clinical, immunophenotypic and genetic features except less frequent expression of BCL6. Regarding expression of CD30 and pSTAT3, only trends were observed (*p* = 0.0727 and *p* = 0.0818, respectively). We believe these results might be due to the relatively low number (*n* = 16) of patients in younger age group because these proteins are more commonly expressed in older age group.

Comparing younger and older patients with EBV+ DLBCL, significant differences were found in median age (43 *vs*. 66 years) and the proportion of patients with IPI > 2 (13% *vs*. 52%). The difference in median age is expected because the comparison was based on age. The IPI score is based on age, stage, serum LDH, ECOG score and number of extranodal sites [14]. Excluding age from the score, 22% of the older patients retained an IPI > 2. The remaining clinical, immunophenotypic, including LMP1 and EBNA2, and genetic features were not different between the two groups. Furthermore, gene expression profiling did not show distinctive features between younger and older group, consistent with data published by others [12]. In a recent study, EBV+ DLBCL in patients aged > 50 years showed overexpression of hsa-miR-126, hsa-miR-146a, hsa-miR-146b, hsa-miR-150 and hsa-miR-222 compared to EBV− DLBCL in the same age group [15]. We compared the expression of these microRNAs in younger versus older patients with EBV+ DLBCL and did not observe any significant differences.

In an earlier report, we showed that EBV infection in DLBCL does not impact survival in Caucasian patients. In this expanded cohort, we reproduced the same result. Separating the younger and older groups, EBV infection in DLBCL also did not correlate with shorter survival in either group. For patients with EBV+ DLBCL, younger and older patients had a similar outcome.

EBV+ DLBCL with CD30 co-expression has extremely poor survival [5] and we reproduced the result in the current study. Considering frequent expression of CD30 in EBV+ DLBCL and availability of brentuximab vedotin, targeting CD30 could be an attractive therapeutic option for patients with EBV+ DLBCL [22].

In summary, about one third of patients with EBV+ DLBCL are younger than 50 years of age and this subgroup has similar clinicopathologic, immunophenotypic features and survival compared with patients who are > 50 years. We also show that the gene expression profiling and microRNA profiles of younger patients with EBV+ DLBCL is similar to older patients. Based on the data present, we suggest that arbitrary age cutoff for EBV+ DLBCL proposed in the WHO classification is unnecessary.

## MATERIALS AND METHODS

### Patient selection

Since our previous report of 28 cases [5], 11 more cases of EBV+ DLBCL were added to the International DLBCL Rituximab-CHOP Consortium Program Study. All cases were reviewed by a group of hematopathologists and were diagnosed according to the WHO criteria. We also identified 7 cases of EBV+ DLBCL by searching the archives of The University of Texas MD Anderson Cancer Center. We classified morphologic variants of EBV+ DLBCL based on the description of Monte-Moreno and colleagues [16]. To compare clinicopathologic and genetic features, cohorts of de novo DLBCL in patients ≤50 years old (*n* = 104) and > 50 years old (*n* = 429) were selected from the Consortium Study. Exclusion criteria included DLBCL transformation from a low-grade B-cell lymphoma, association of immunodeficiency (e.g., HIV infection or common variable immunodeficiency), primary cutaneous DLBCLs, primary central nervous system DLBCLs, and primary mediastinal large B-cell lymphomas. This study was conducted in accord with the Declaration of Helsinki and was approved by the Institutional Review Boards (IRB) of all participating collaborative institutions. The overall study was approved by the IRB at The University of Texas MD Anderson Cancer Center.

### Immunohistochemistry and in situ hybridization methods

Hematoxylin and eosin stained slides from each case were reviewed and tumor-rich areas were selected. Tissue microarrays (TMA) were constructed using a tissue microarrayer (Beecher Instrument, Silver Spring, MD). Immunohistochemical analysis was performed on 4-μm TMA sections using a streptavidin-biotin complex technique with antibodies reactive with the following antigens: BCL2, BCL6, CD10, CD30, EBNA2, FOXP-1, GCET1, IRF4/MUM-1, LMP1, MYC, NF-ĸB p50, p65 and c-Rel, pSTAT3, and p53. *In situ* hybridization (ISH) for EBV-encoded RNA (EBER) was performed. Due to tissue exhaustion, staining was not always available for each marker. Antigen expression was scored in 10% increments by assessing the percentage of immunoreactive tumor cells. Receiver-operating characteristic (ROC) curve analyses and X-tile analyses were used to determine a prognostically relevant cutoff with optimal sensitivity and specificity for each marker [17]. When an optimal cutoff value for an individual marker could not be determined by ROC curve, a conventional cutoff value was decided based on reports in the literature. The cutoff scores for these markers used in this study were as follows: 10% for EBER, EBNA2 and LMP1; 20% for CD30, p50 and p53; 30% for CD10, BCL6 and c-Rel; 40% for MYC and p65; 50% for pSTAT3; 60% for GCET1, MUM-1 and FOXP1; and 70% for BCL2.

### Fluorescence *in situ* hybridization (FISH)

FISH analysis was performed on formalin-fixed, paraffin embedded tissue sections using BCL2 and BCL6 dual-color break-apart probes (Vysis), *MYC* locus-specific *IGH/MYC/CEP8* tricolor dual-fusion probes, and a locus-specific MYC dual-color break-apart probe (Vysis) as described previously [18]. *TP53* sequencing was performed using extracted genomic DNA from formalin-fixed, paraffin-embedded tissue in the training set. The coding sequence (exons 2-11) and splicing sites were sequenced using p53 AmpliChip (Roche Molecular Systems) as described previously [19]. For data analysis, the *TP53* reference sequence (NC_000017.10) in the GenBank database was used.

### Gene expression profiling

Total RNA was extracted from 25 formalin-fixed, paraffin-embedded (FFPE) tissue blocks using the High Pure RNA Extraction Kit (Roche Applied Science) and subjected to gene expression profiling (GEP) as has been described [20]. For data analysis and classification, we used the DQN algorithm, which is the noncentral trimmed mean of differences between perfect match and mismatch intensities with quantile normalization [21]. DQN was normalized with beta distribution and a Bayesian model was used to determine the classification probability.

### Cell-of-origin (COO) classification

Cell-of-origin classification was achieved by combining GEP (considered the “gold standard”) and IHC data as described previously [20]. In a total of 41 cases COO was determined, by GEP with 25 cases and by IHC in 8 cases, respectively.

### EBV+ DLBCL microRNA profiling

The HTG EdgeSeq Whole Transcriptome Assay coupled with the Illumina HiSeq was used for measuring expression of hsa-miR-126, hsa-miR-146a, hsa-miR-146b, hsa-miR-150 and hsa-miR-222 from FFPE tissue blocks. A total of 9 patients were tested including 2 younger (≤50 years) and 7 older (> 50 years) patients with EBV+ DLBCL. Selection of 5 microRNAs was based on previously published data.[15]

### Statistical analysis

Clinical and laboratory features were compared with the Fisher exact test for categorical variables and Mann–Whitney U test or unpaired t-test if for continuous variables. Overall survival (OS) and progression-free survival (PFS) were defined from the date of diagnosis to the date of last follow-up or death and from the date of diagnosis to the date of progression or death, respectively. Survival distributions were estimated with the Kaplan–Meier method, with difference compared by the log-rank test. Univariate analysis was performed using the Cox proportional hazards regression model. Two-sided *p* < 0.05 was considered to be statistically significant. GraphPad Prism V5 (La Jolla, CA) and SPSS Statistics V21 (Armonk, NY) were used for statistical analyses.
